# Quantifying ‘Causality’ in Complex Systems: Understanding Transfer Entropy

**DOI:** 10.1371/journal.pone.0099462

**Published:** 2014-06-23

**Authors:** Fatimah Abdul Razak, Henrik Jeldtoft Jensen

**Affiliations:** 1 Complexity & Networks Group and Department of Mathematics, Imperial College London, London, United Kingdom; 2 School of Mathematical Sciences, Faculty of Science & Technology, Universiti Kebangsaan Malaysia, Bangi, Selangor, Malaysia; University of Namur, Belgium

## Abstract

‘Causal’ direction is of great importance when dealing with complex systems. Often big volumes of data in the form of time series are available and it is important to develop methods that can inform about possible causal connections between the different observables. Here we investigate the ability of the Transfer Entropy measure to identify causal relations embedded in emergent coherent correlations. We do this by firstly applying Transfer Entropy to an amended Ising model. In addition we use a simple Random Transition model to test the reliability of Transfer Entropy as a measure of ‘causal’ direction in the presence of stochastic fluctuations. In particular we systematically study the effect of the finite size of data sets.

## Introduction

Many complex systems are able to self-organise into a critical state [1], [2]. The local properties of the system will typically fluctuate in time and space but the way the fluctuations are interrelated or correlated may differ. In this context a critical state is defined in terms of the way in which the correlations of the local fluctuations decay in space and time. When a system isn't critical, the correlations of the fluctuations of a quantity 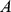 measured in two different positions at two different times, say 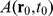 and 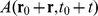 decay as an exponential function of the separation in space 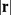 and also decay exponentially as function of the separation in time 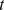. However in a critical state the correlations exhibit a much slower algebraic decay, i.e. the correlation functions decay as negative powers of 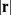 and 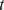. This is the behaviour observed at second order phase transitions in thermal equilibrium, which are denoted the critical points. The slow algebraic decay of correlations is equivalent to correlations effectively spanning across the entire system. Or in other words, in the critical state local distortions can propagate throughout the entire system [2]–[4]. We address here how to identify directed stochastic causal connections embedded in a background of strongly correlated stochastic fluctuations.

Most of ‘causality’ and directionality measures have been tested on low dimension systems and neglect addressing the behaviour of systems consisting of large numbers of interdependent degrees of freedom that is a main feature of complex systems. From a complex systems point of view, on one hand there is the system as a whole (collective behaviour) and on another there are individual interactions that lead to the collective behaviour. A measure that can help understand and differentiate these two elements is needed. We shall first seek to make a clear definition of ‘causality’ and then relate this definition to complex systems. We outline the different approaches and measures used to quantify this type of ‘causality’. We highlight that for multiple reasons, Transfer Entropy seems to be a very suitable candidate for a ‘causality’ measure for complex systems. Consequently we seek to shed some light on the usage of Transfer Entropy on complex systems.

To improve our understanding of Transfer Entropy we study two simplistic models of complex systems which in a very controllable way generate correlated time series. Complex system whose main characteristic consist in essential cooperative behaviour [5] takes into account instances when the whole system is interdependent. Therefore, we apply Transfer Entropy to the (amended) Ising model in order to investigate its behaviour at different temperatures particularly near the critical temperature. Moreover, we are also interested in investigating the different magnitude of Transfer Entropy in general (which is not fully understood [6]) by looking at the effect of different transition probabilities, or activity levels. We discuss the interpretation of the different magnitudes of the Transfer Entropy by varying transition rates in a Random Transition model.

## Quantifying ‘Causality’

The quantification of ‘causality’ was first envisioned by the mathematician Wiener [7] who propounded the idea that the ‘causality’ of a variable in relation to another can be measured by how well the variable helps to predict the other. In other words, variable 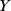 ‘causes’ variable 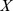 if the ability to predict 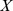 is improved by incorporating information about 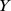 in the prediction of 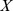. The conceptualisation of ‘causality’ as envisioned by Wiener was formulated by Granger [8] leading to the establishment of the Wiener-Granger framework of ‘causality’. This is the definition of ‘causality’ that we shall adopt in this paper.

In literature, references to ‘causality’ take many guises. The term directionality, information transfer and sometimes even independence can possibly refer to some sort of ‘causality’ in line with the Wiener-Granger framework. Continuing the assumption that 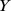 causes 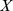, one would expect the relationship between 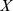 and 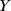 to be asymmetric and that the information flows in a direction from the source 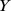 to the target 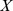. One can assume that this information transfer is the unique information provided by the causal variable to the affected one. When one variable causes another variable, the affected variable (the target) will be dependent (to certain extent) on the causal variable (the source). There must exist a certain time lag however small between the source and the target [9]–[11], this will be henceforth referred to as the causal lag [8]. One could also say the Wiener-Granger framework of prediction based ‘causality’ is equivalent to looking for dependencies between the variables at a certain causal lag.

Roughly, there are two different approaches in establishing ‘causality’ in a system. One approach is to make a qualified guess of a model that will fit the data, called the confirmatory approach [12]. Models of this nature are typically very field specific and rely on particular insights into the mechanism involved. A contrasting approach known as the exploratory approach, infers ‘causal’ direction from the data. This approach does not rely on any preconceived idea about underlying mechanisms and let results from data shape the directed model of the system. Most of the measures within the Wiener-Granger framework falls into this category. One can think of the different approaches as being on a spectrum from purely confirmatory to purely exploratory.

The nature of complex systems calls for the exploratory approach. The abundance of data emphasises this even more so. In fact ‘causality’ measures in the Wiener Granger framework have been increasingly utilised on data sets obtained from complex systems such as the brain [13], [14] and financial systems [15]. Unfortunately, most of the basic testings of the effectiveness of these measures are mostly done on dynamical systems [16]–[18] or simple time series, without taking into account the emergence of collective behaviour and criticality. Complex systems are typically stochastic and thus different from deterministic systems where the internal and external influences are distinctly identified. As mentioned above, here we focus on the emergence of collective behaviour in complex systems and in particular on how the intermingling of the collective behaviour with individual (coupled) interactions complicates the identification of ‘causal’ relationships. Identifying a measure that is able to distinguish between these different interactions will obviously help us to improve our understanding of the dynamics of complex systems.

## Transfer Entropy

Within the Wiener-Granger framework, two of the most popular ‘causality’ measure are Granger Causality (G-causality) and its nonlinear analog Transfer Entropy. G-causality and Transfer Entropy are exploratory as their measures of causality are based on distribution of the sampled data. The standard steps of prediction based ‘causality’ that underlies these measures can be summarized as follows. Say we want to test whether variable 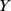 causes variable 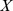. The first step would be to predict the current value of 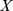 using the historical values of 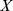. The second step is to do another prediction where the historical values of 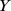 and 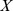 are both used to predict the current value of 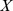. And the last step would be to compare the former to the latter. If the second prediction is judged to be better than the first one, then one can conclude that 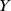 causes 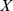. This being the main idea, we outline why Transfer Entropy is more suitable for complex systems.

Granger causality is the most commonly used ‘causality’ indicator [9]. However, in the context of the nonlinearities of a complex systems (collective behaviour and criticality being the main example), using G-causality may not be sufficient. Moreover, the inherently linear autoregressive framework makes G-causality less exploratory than Transfer Entropy. Transfer Entropy was defined [16], [17] as a nonlinear measure to infer directionality using the Markov property. The aim was to incorporate the properties of Mutual Information and the dynamics captured by transition probabilities in order to understand the concept and exchange of information. More recently, the usage of Transfer Entropy to detect causal relationships [19]–[21] and causal lags (the time between cause and effect) has been further examined [6], [22]. Thus we are especially interested in Transfer Entropy due to its propounded ability to capture nonlinearities, its exploratory nature as well as its information theoretic background that provides information transfer related interpretation. Unfortunately, some of the vagueness in terms of interpretation may cause confusion in complex systems. The rest of the paper is an attempt to discuss these issues in a reasonably self-contained manner.

### Mutual Information based measures

Define random variables 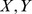 and 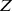 with discrete probability distributions 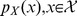, 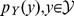 and 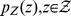. The entropy of 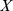 is defined [23], [24] as
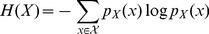
(1)where log to the base 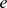 and 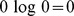 is used. The joint entropy of 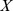 and 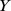 is defined as
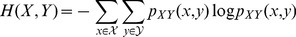
(2)and the conditional entropy can be written as
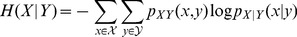
(3)where 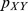 is the joint distribution and 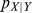 is the respective conditional distribution. The Mutual Information [24], [25] is defined as
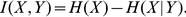
(4)Taking into account conditional variables, the conditional Mutual Information [19], [24] is defined as 
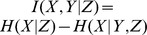
. A variant of conditional Mutual Information namely the Transfer Entropy was first defined by Schreiber in [16]. Let 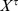 be the variable 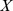 that is shifted by 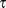, so that the values of 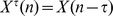 where 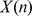 is the value of 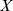 at time step 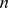 and similarly for 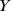. We highlight a simple form of Transfer Entropy where conditioning is minimal such that
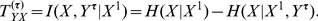
(5)The idea is that, if 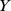 causes 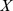 at causal lag 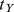, then 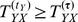 for any lag 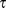 since 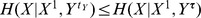 due to the fact that 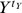 should provide the most information about the change of 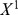 to 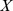. This simple form allows us to vary the values of time lag 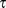 in ascertaining the actual causal lag. This form of Transfer Entropy was also used in [13], [18], [22], [26], [27]. The Transfer Entropy in equation (5) can also be written as
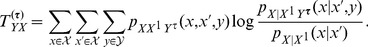
(6)Our choice of this simple definition was motivated by the fact that it directly captures how the state of 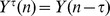 influences the changes in 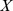 i.e. from 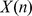 to 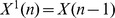. In other words, equation (5) is tailor made to measure whether the state of 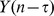 influences the current changes in 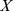. This coincides with the predictive view of ‘causality’ in the Wiener-Granger framework where the current state of one variable (the source) influences the changes in another variable (the target) in the future. The same concept will be applied in order to probe this kind of ‘causality’ in our models.

## The Ising Model

A system is critical when correlations are long ranged. A simple prototype example is the Ising model [2] at critical temperature, 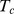. Away from 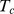 correlations are short ranged and dies off exponentially with separation. We shall apply Transfer Entropy to the Ising model in order to investigate its behaviour at different temperatures particularly in the vicinity of the critical temperature. One can visualize the 2D Ising model as a two dimensional square lattice with length 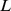 composed of 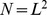 sites 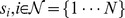. These sites can only be in two possible states, spin-up (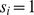) or spin-down (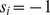). We restrict the interaction of the sites to only its nearest neighbours (in two dimensions this will be sites to the north, south, east and west). Let the interaction strength between 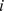 and 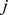 be denoted by
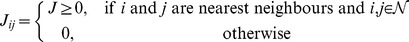
(7)so that the Hamiltonian (energy), 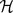, is given by [2], [28]

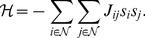
(8)

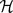
 is used to obtain the Boltzmann (Gibbs) distribution 
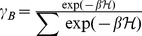
 with 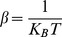 where 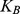 is the Boltzmann constant and 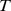 is temperature.

We implement the usual Metropolis Monte Carlo (MMC) algorithm [2], [29], [30] for the simulation of the Ising model in two dimensions with periodic boundary conditions. The algorithm proposed by Metropolis and co-workers in 1953 was designed to sample the Boltzmann distribution 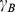 by artificially imposing dynamics on the Ising model. The implementation of the MMC algorithm in this paper is outlined as follows. A site is chosen at random to be considered for flipping (change of state) with probability 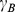. The event of considering the change and afterwards the actual change (if accepted) of the configuration, shall henceforth be referred to as flipping consideration. A sample is taken after each 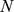 flipping considerations. The logic being that, since sites to be considered are chosen randomly one at a time, after 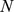 flips, each site will on average have been selected for consideration once. The interaction strength is set to be 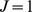 and the Boltzmann constant is fixed as 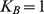 for all the simulations. We let the system run up to 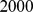 samples before sampling at every 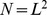 time steps.

Through the MMC algorithm, a Markov chain (process) is formed for every site on the lattice. The state of each site at each sample will be taken as a time step 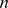 in the Markov chain 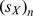. Let 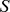 be the number of samples (length of the Markov chains). To get the probability values for each site, we utilise temporal average. All the numerical probabilities obtained for the Ising model in this paper have been obtained by averaging over simulations with 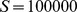 unless stated otherwise.

### Measures on Ising model

**Figure 1:**
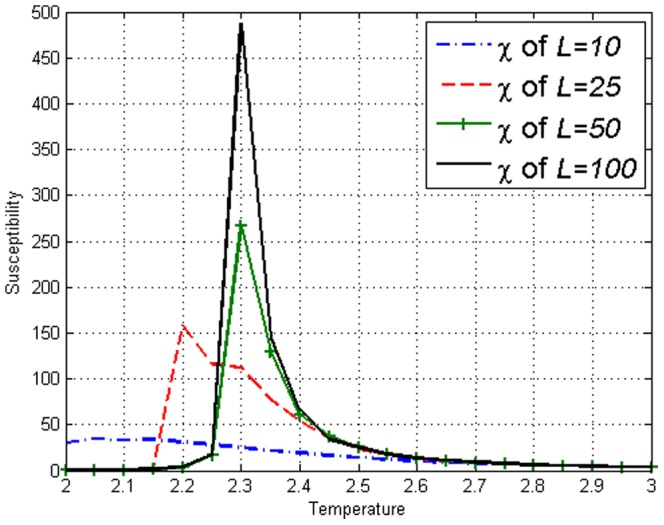
Susceptibility 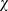 on the Ising model with lengths *L*=10,25,50,100 obtained using equation (9). Peaks can be seen at respective 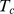.

In an infinite two dimensional lattice, the phase transition of the Ising model with 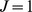 and 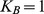 is known to occur at the critical temperature 
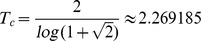
 [2]. In a finite system, due to finite size effects, the critical values will not be quite as exact, we will call the temperature where the transition effectively occurs in the simulation as the crossover temperature 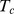. Susceptibility 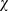 is an observable that is normally used to identify 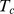 for the Ising model as seen in Figure (1). In order to define 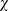, let 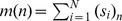 be the sum of spins on a lattice of size 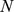 at time steps 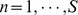. The susceptibility [2] is given by
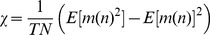
(9)where 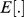 is the expectation in terms of temporal average and 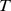 is temperature. The covariance on the Ising model can be defined as
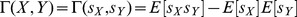
(10)where 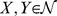.

**Figure 2:**
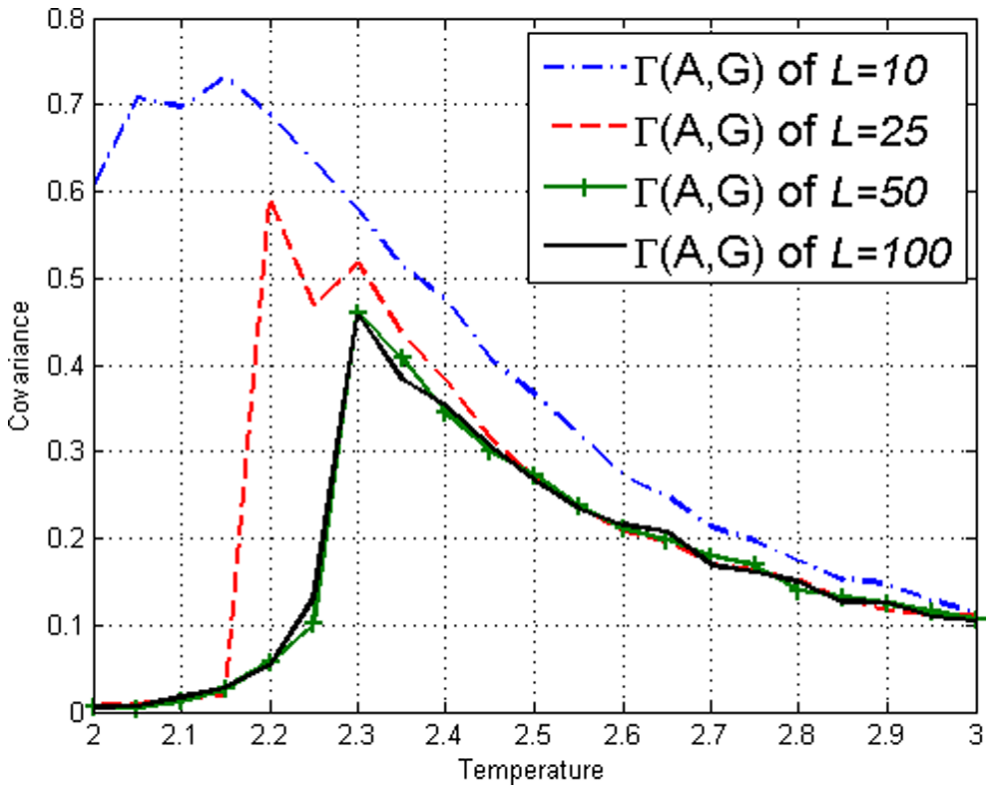
Covariance 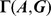 on the Ising model with lengths *L*=10,25,50,100 obtained using equation (10).

**Figure 3:**
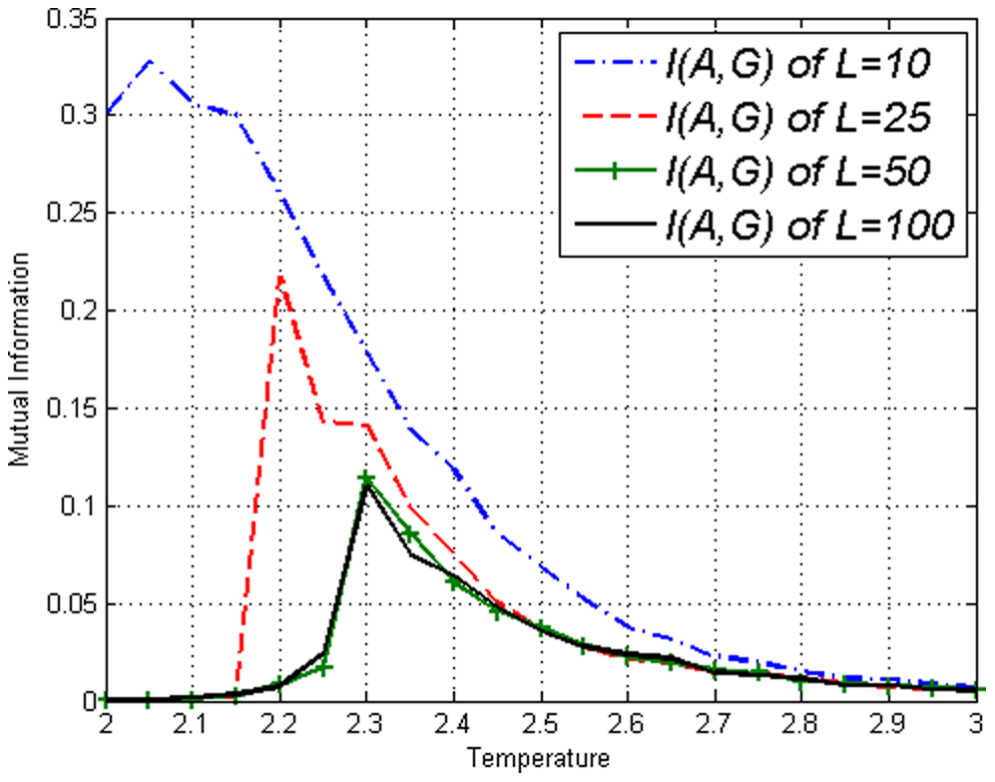
Mutual Information 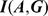 on the Ising model with lengths *L*=10,25,50,100 obtained using equation (4).

To display measures applied on individual sites, let sites 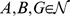 represent coordinates 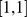, 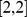 and 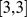 respectively. The values of the covariance 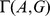 and 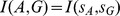 is displayed in Figure (2) and Figure (3). It can be seen that for the Ising model, Mutual Information gives no more information than covariance. From this figure, one can see that the values are system size dependent up to system size 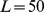 or 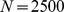. We conclude from this, that up to this length scale, correlations are detectable across the entire lattice [2]. Thus we shall frequently utilize 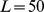 when illustration is required.

**Figure 4:**
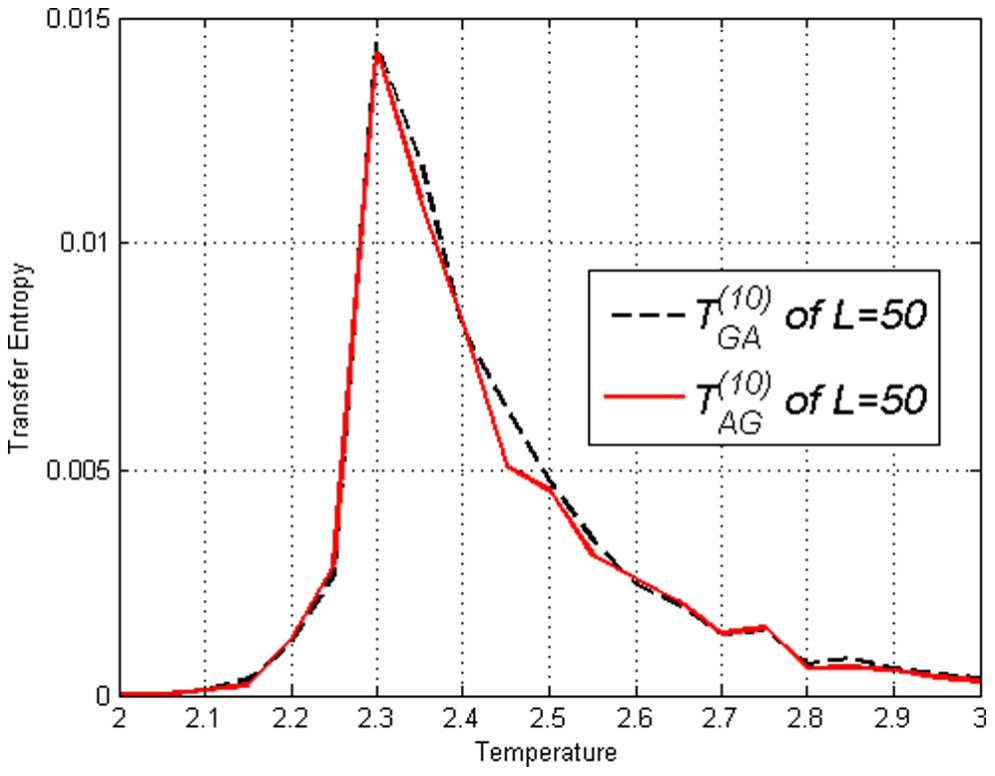
Transfer Entropy 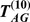 and 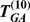 on the Ising model of lengths *L*=50 obtained using equation (5). Peaks for both direction are at 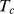.

**Figure 5:**
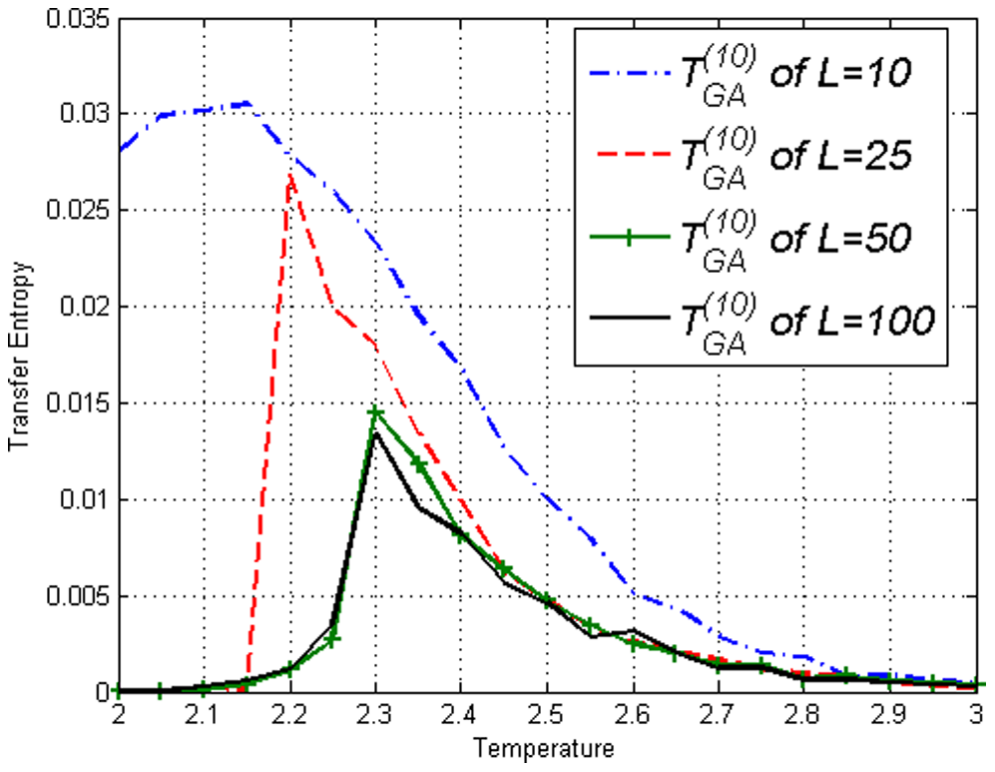
Transfer Entropy 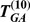 on the Ising model of lengths *L*=10,25,50,100 obtained using equation (5). Peaks can be seen at respective 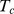.

**Figure 6:**
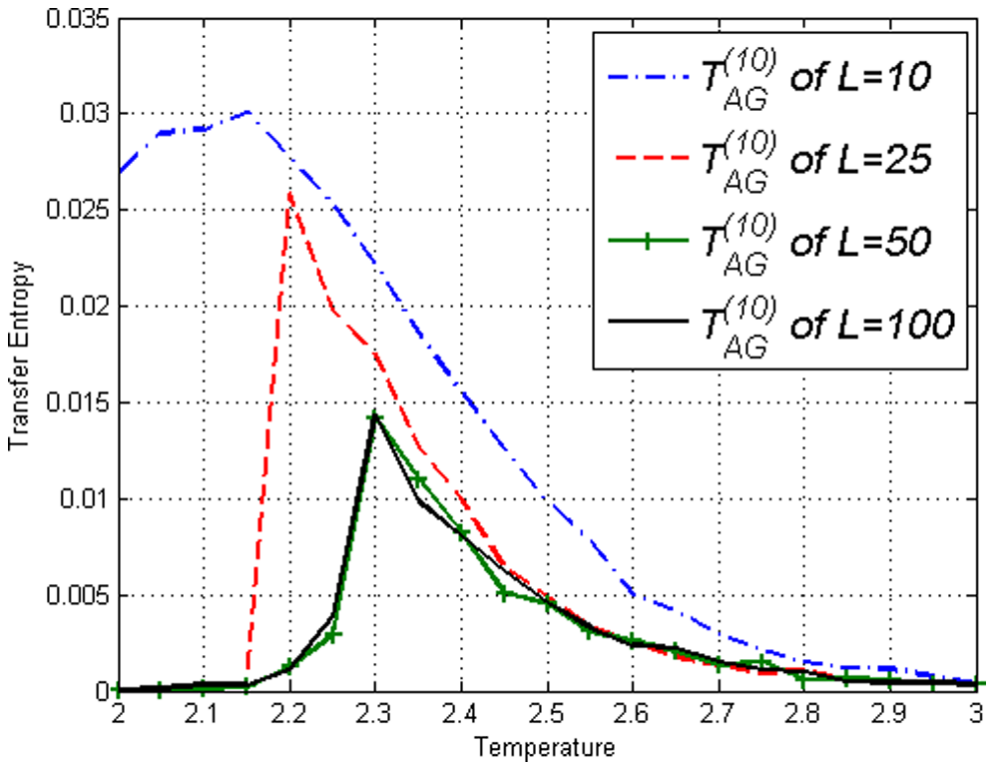
Transfer Entropy 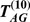 on the Ising model of lengths *L*=10,25,50,100 obtained using equation (5). Peaks can be seen at respective 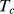.

Using time shifted variables we obtained the Transfer Entropy 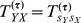 in Figures (4–6). By looking at Figure (4) and then contrasting Figures (5) and (6), one can see that there is no clear difference between 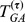 and 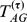 in the figures thus no direction of ‘causality’ can be established between 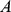 and 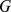. This is expected due to the symmetry of the lattice. More interestingly, the fact that Transfer Entropy peaks near 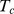 can be due to the fact that at 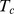 the correlations span across the entire lattice. Therefore, one may say that the critical transition and collective behaviour in the Ising model is detected by Transfer Entropy as a type of ‘causality’ that is symmetric in both directions. It is logical to interpret collective behaviour as a type of ‘causality’ in all directions since information is disseminated throughout the whole lattice when it is fully connected. This is an important fact to take into account when estimating Transfer Entropy on complex systems.

## Amended Ising Model

In the amended Ising model we introduce an explicit directed dependence between the sites 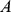, 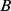 and 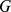 in order to study how well Transfer Entropy is able to detect this causality. We will define the amended Ising model using the algorithm outlined as follows. At each step in the algorithm a site chosen at random will be considered for flipping with a certain probability 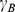 except when 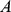 or 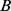 is selected where an extra condition needs to be fulfilled first before it can be allowed to change (flip). If 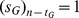, 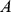 (or 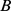) can be considered for flipping with probability 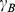 as usual, however if 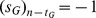, no change is allowed. Thus only one state of 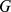 (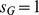 in this case) allows sites 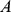 and 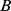 to be considered for flipping. Therefore, although 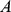 and 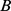 have their own dynamics, their changes still depend on 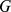.

**Figure 7:**
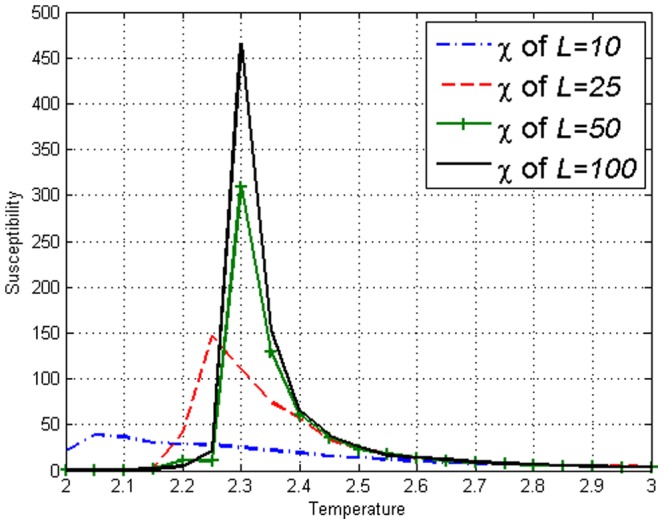
Susceptibility 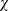 on the amended Ising model of lengths *L*=10,25,50,100 obtained using equation (9). Peaks can be seen at respective 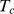.

**Figure 8:**
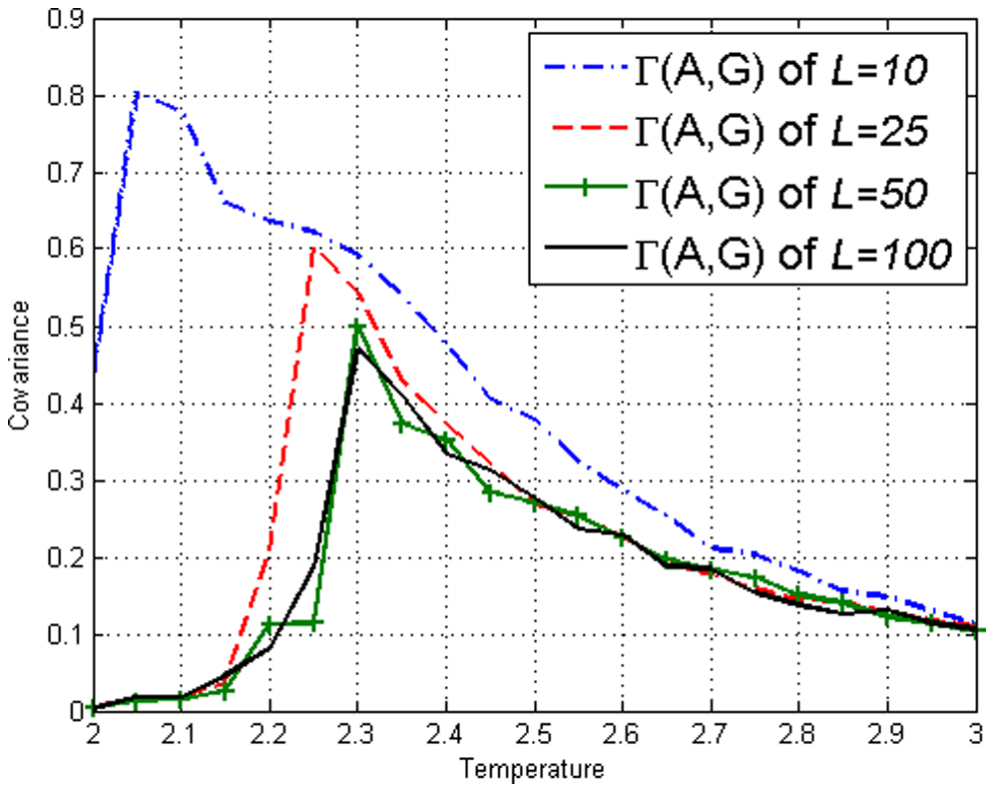
Covariance 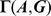 on the amended Ising model of lengths *L*=10,25,50,100 obtained using equation (10). Peaks can be seen at respective 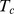, similar to Figure (2) of the Ising model.

**Figure 9:**
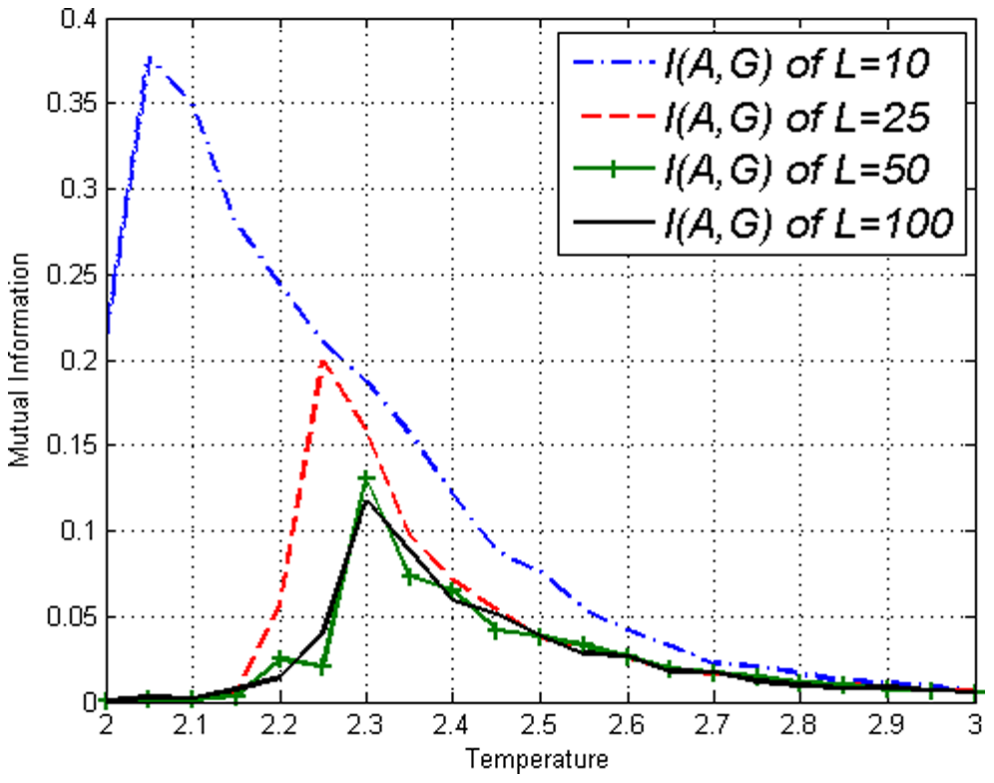
Mutual Information 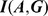 on the amended Ising model with lengths *L*=10,25,50,100 obtained using equation (4). Not much different from results on the Ising model in Figure 3.

**Figure 10:**
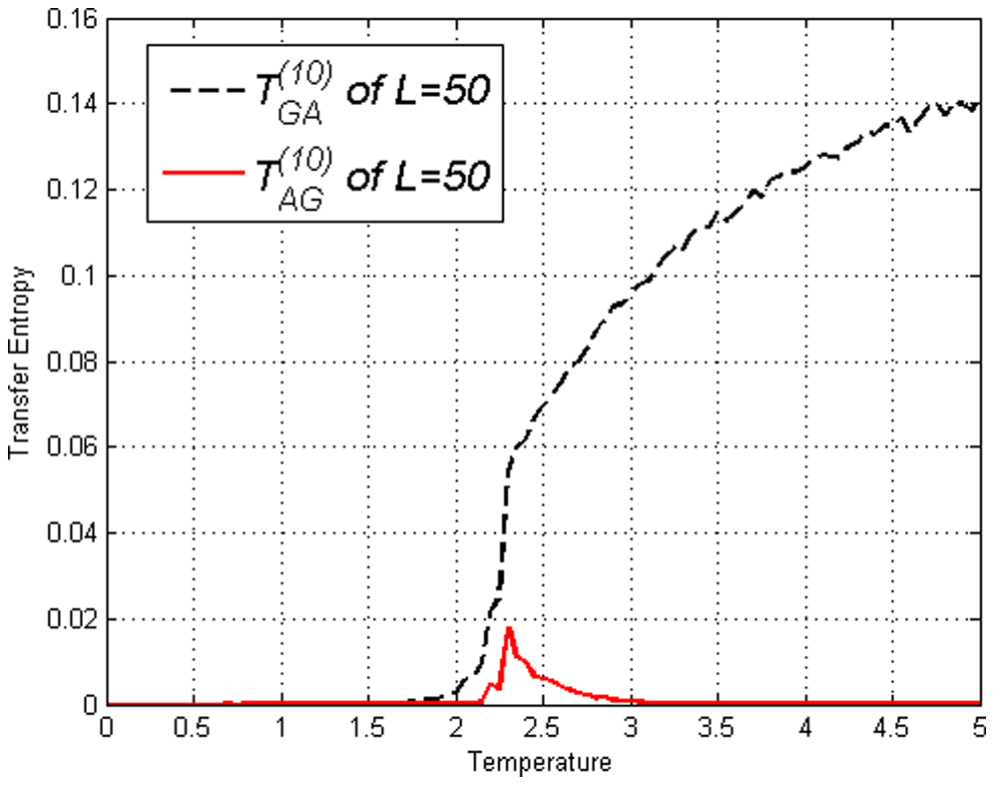
Transfer Entropy 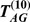 and 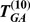 on the amended Ising model of lengths 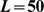 and 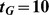, obtained using equation (5). Direction 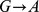 at time lag 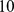 is indicated. Very different from result on Ising model in Figure 4.

**Figure 11:**
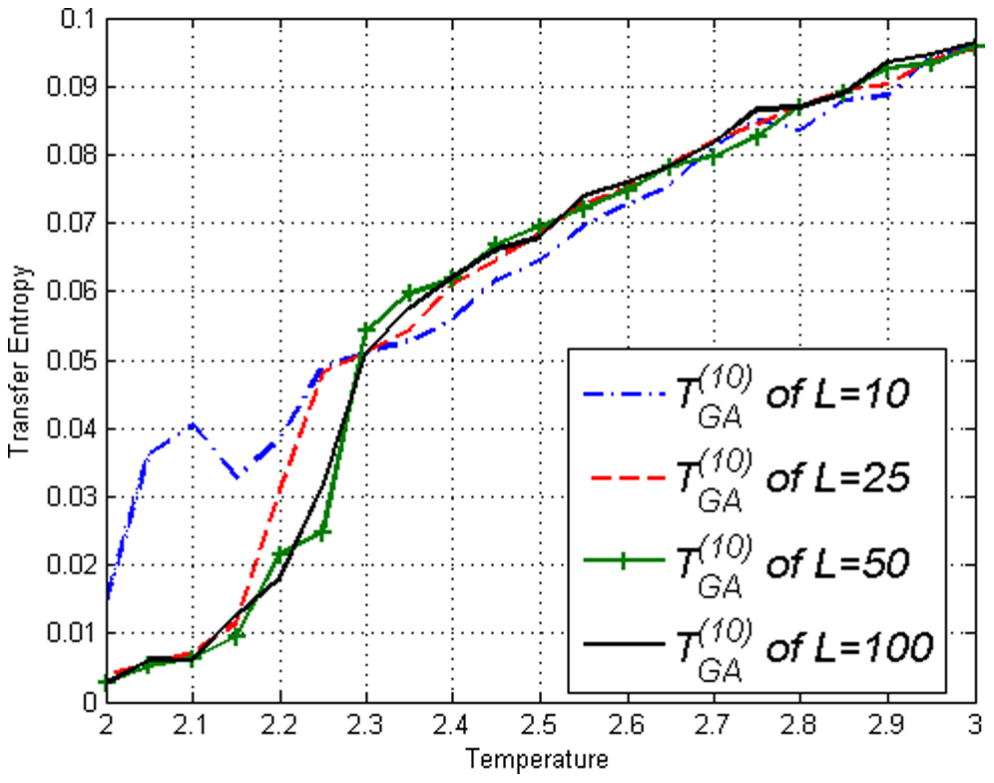
Transfer Entropy 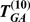 on the Ising model of lengths *L*=10,25,50,100 obtained using equation (5). Values continue to increase after 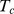 which is very different from Figure (5).

**Figure 12:**
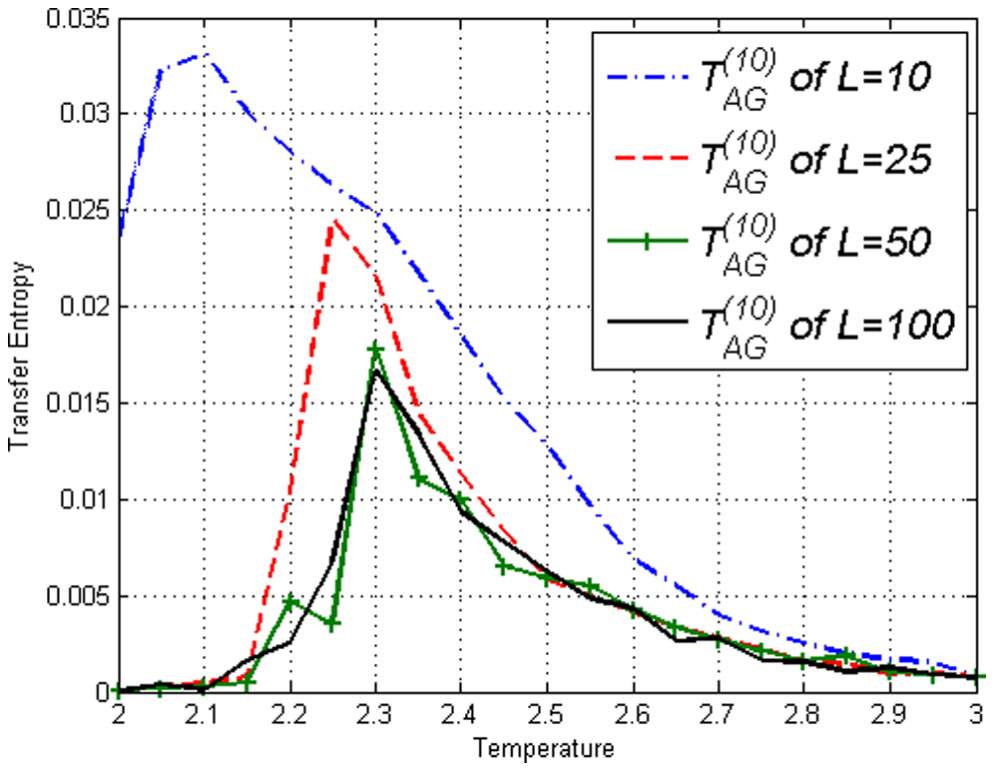
Transfer Entropy 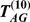 on the Ising model of lengths *L*=10,25,50,100 obtained using equation (5). Peaks can be seen at respective 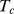, similar to Ising model results in Figure (6).

We simulated the amended Ising model with 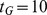 for different lattice lengths 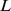. Figures (7) display the values of susceptibility 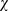 on the model and the peaks clearly show the presence of 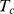 in our model just like Figure (1) of the Ising model. Figures (8) and (9) display the values of the covariance 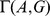 and the Mutual Information 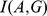 respectively. We reiterate that our correlations reach across the system for 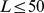 [2], [31]. While covariance and Mutual Information gives similar results to those of the standard Ising model as in Figures (2) and (3), a difference is clearly seen in Transfer Entropy values. Figure (10–12) displays the contrasts of 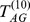 and 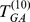 on the amended Ising model which explicitly indicates the direction of ‘causality’ 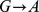. While Figure (12) is not very different from Figure (6), Figures (10) and (11) are clearly different from their counterparts in the Ising model, Figures (4) and (5). Transfer Entropy captures the effect of the amendment.

**Figure 13:**
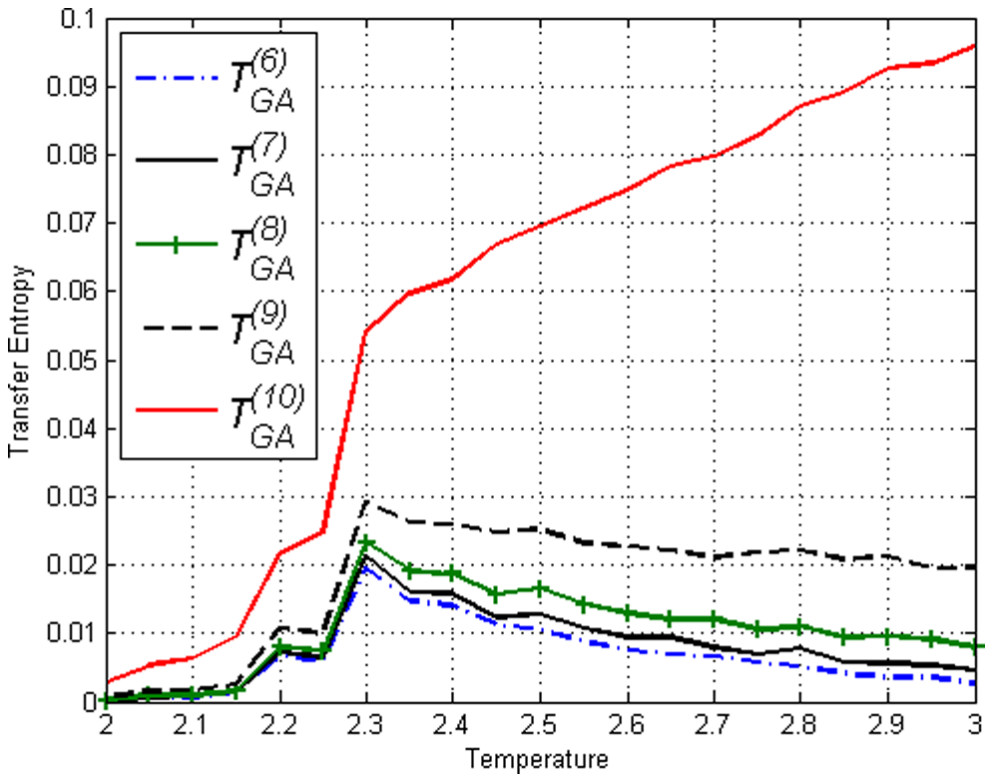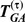 versus 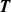 for different time lags 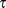 in amended Ising model with 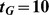 and 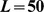 using equation (5). The figure shows the effect of separation in time.

**Figure 14:**
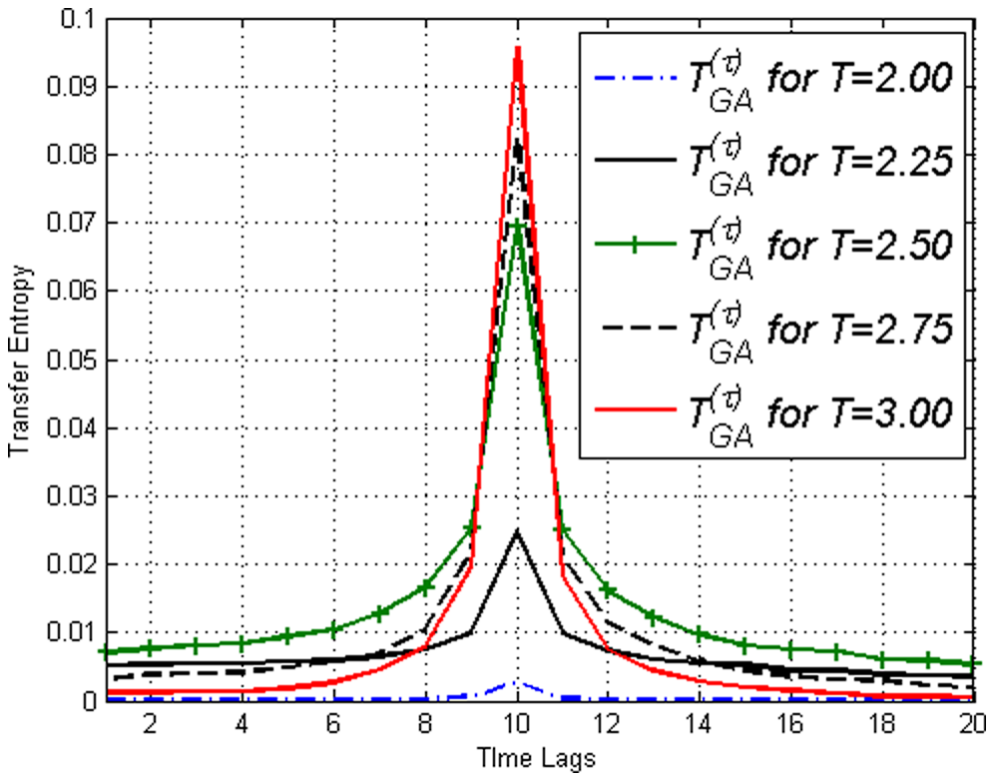
A different view of Figure (13) where 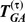 versus 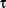 for different temperatures 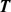 is plotted instead. 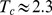
. Figure highlights time lag detection.

Furthermore with this amendment, one can utilize Transfer Entropy to illustrate the effect of separation in time. The effect of deviation from the predetermined causal lag 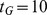, can be clearly seen in Figure (13), where the values of 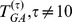 reduces to 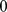 but at different rates depending on the deviation of 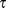 from 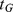. The further away from 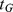, the faster the decrease to 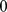. Figure (14) is simply Figure (13) plotted over different time lags 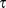 to illustrate how Transfer Entropy correctly and distinctly identified causal lag 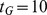.

**Figure 15:**
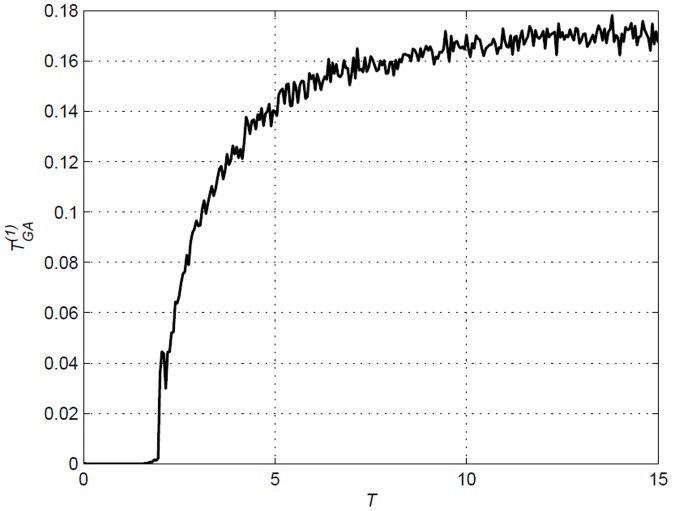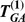 in Figure 17 up to 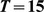. Transfer Entropy stabilizes due to Boltzmann distribution that approaches uniform distribution at higher temperatures.

That temperature is a main factor in influencing the strength of Transfer Entropy values is apparent in all the figures in this section. One can observe, especially in Figure (13), that the Transfer Entropy values approaches 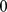 as they get further away from 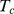 except when the time lag 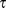 matches the delay induced (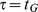), in which case the Transfer Entropy value stabilizes to a certain fixed value as seen in Figure (15). In the vicinity of 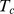, the lattice is highly correlated thus subsequently leading to higher values of Transfer Entropy. The increase and value stabilization after 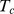 is due to the fact that, as temperature increases, the probability for all ‘flipping considerations’ approaches a uniform distribution. This leads to transfer of information between site 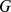 and sites 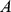 and 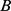 occurring much more frequently at elevated temperature.

**Figure 16:**
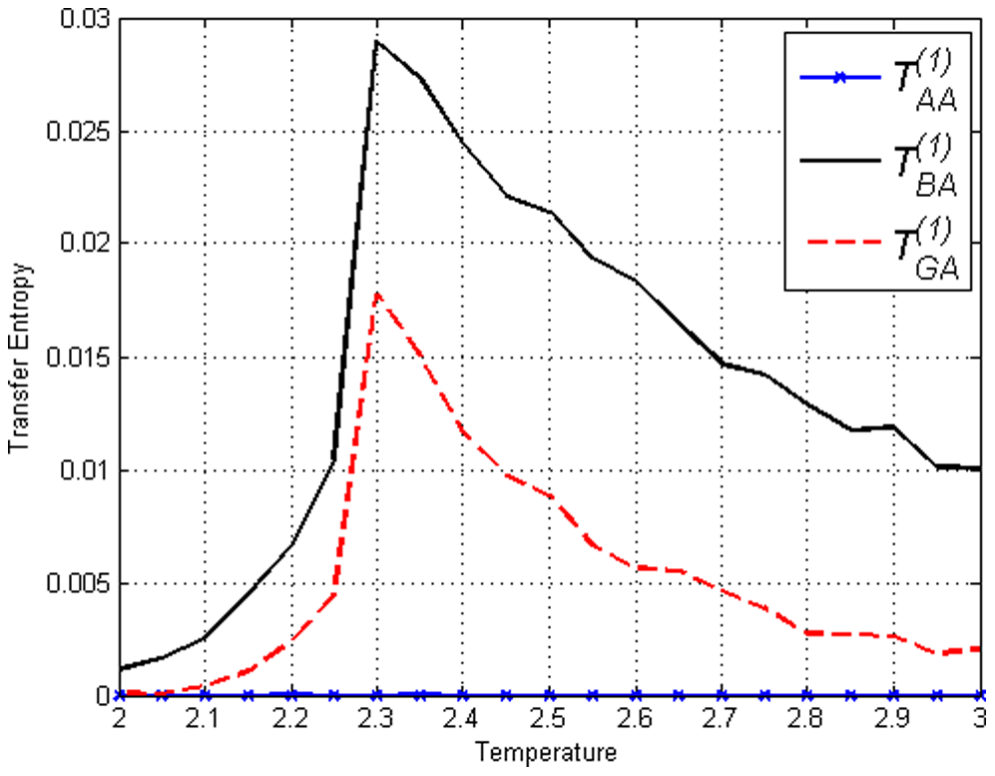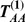, 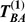 and 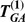 in the Ising model with 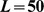. 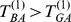
 due to distance (separation) in space where 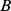 is closer to 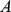 than 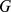. The nearest neighbour effect is observed.

**Figure 17:**
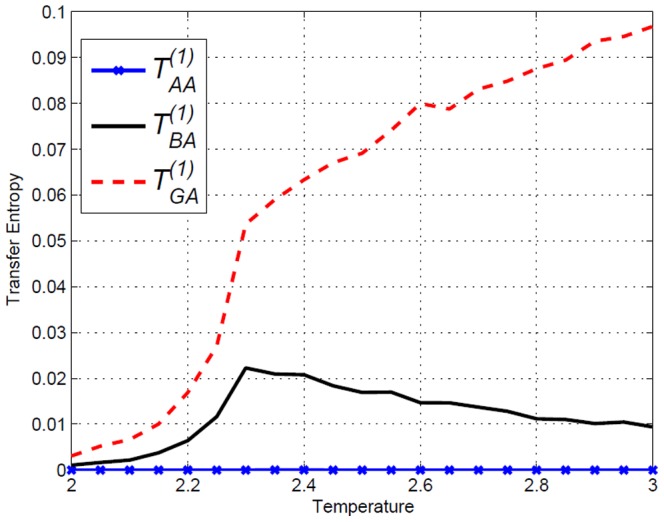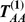, 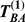 and 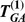 in the amended Ising model with 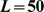 and 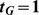. 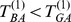
 due to implanted ‘causal’ lag. The effect of separation in space is no longer visible.

Figure (16) and (17) display Transfer Entropy values for the Ising model and amended Ising model with 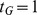 respectively. The figures illustrate the mechanism in which Transfer Entropy detects the predefined causal delay. Consider the following question: which site ‘causes’ site 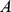
? Firstly we see that 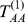 is zero in both figures due to the definition in equation (5). Note that by our definition this is only for 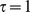, if 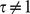 the Transfer Entropy value will be nonzero and also peak at 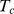. More importantly we see that 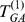 is different from 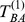. In Figure (16) of the Ising model, the difference is due to separation (distance) in space and nearest neighbour interaction in the model, thus 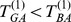 since 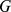 is further away from 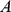 than 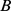. But in Figure (17) of the amended Ising model, the opposite is true and separation in space does not dominate the Transfer Entropy value in this interaction. The figure very clearly indicates that 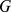 ‘causes’ 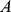 at 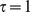 and 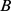 does not. In other words, in the amended Ising model Transfer Entropy identifies 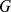 as a source in which one of the target is 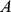, whereas in the Ising model the expected nearest neighbour dynamics presides. This result is only obtained for measures sensitive to transition probabilities. Measures that depend only on static probabilities such as covariance, Mutual Information and conditional Mutual Information will only give values in accordance to the underlying nearest neighbour dynamics in both the Ising model and the amended Ising model [32].

### Transfer Entropy, directionality and change

**Figure 18:**
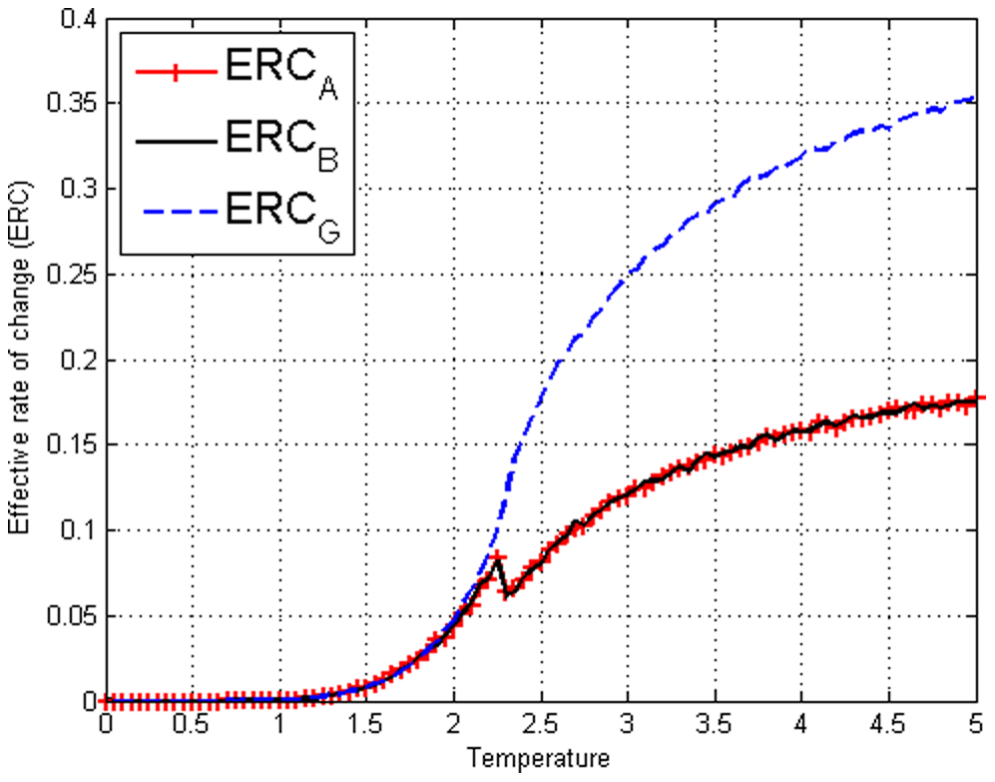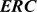 (Expected rate of change) of sites 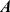, 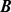 and 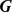 on amended Ising model with 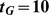 and 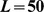.

**Figure 19:**
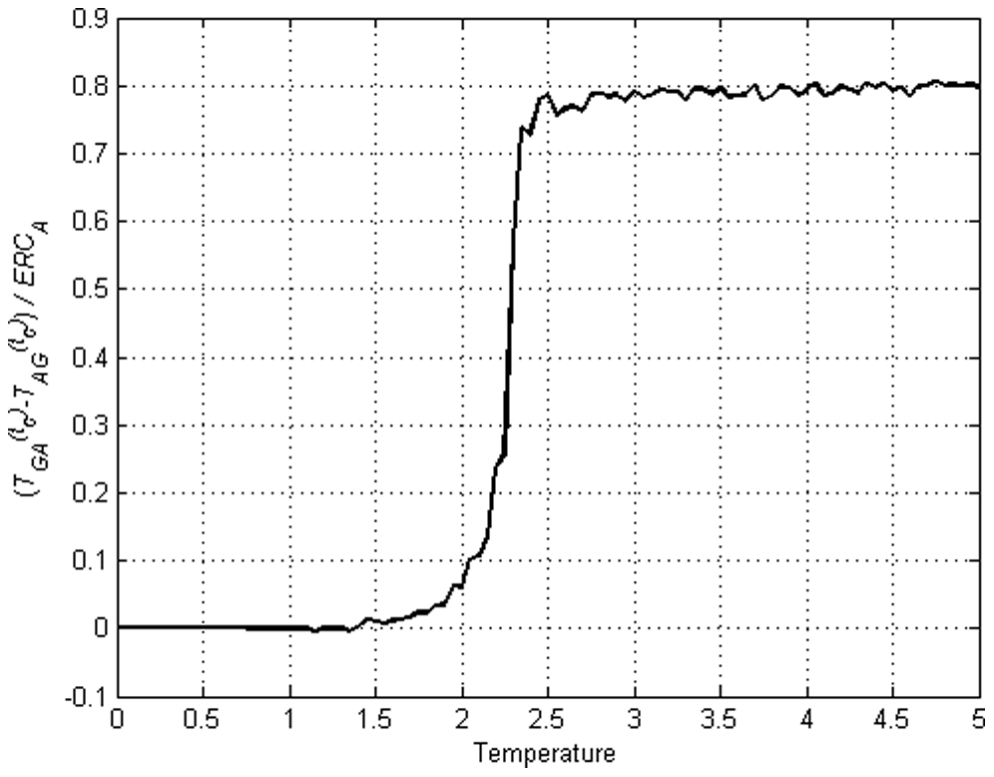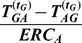 on amended Ising model with 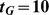 and 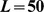 displaying phase-transition like behaviour.

**Figure 20:**
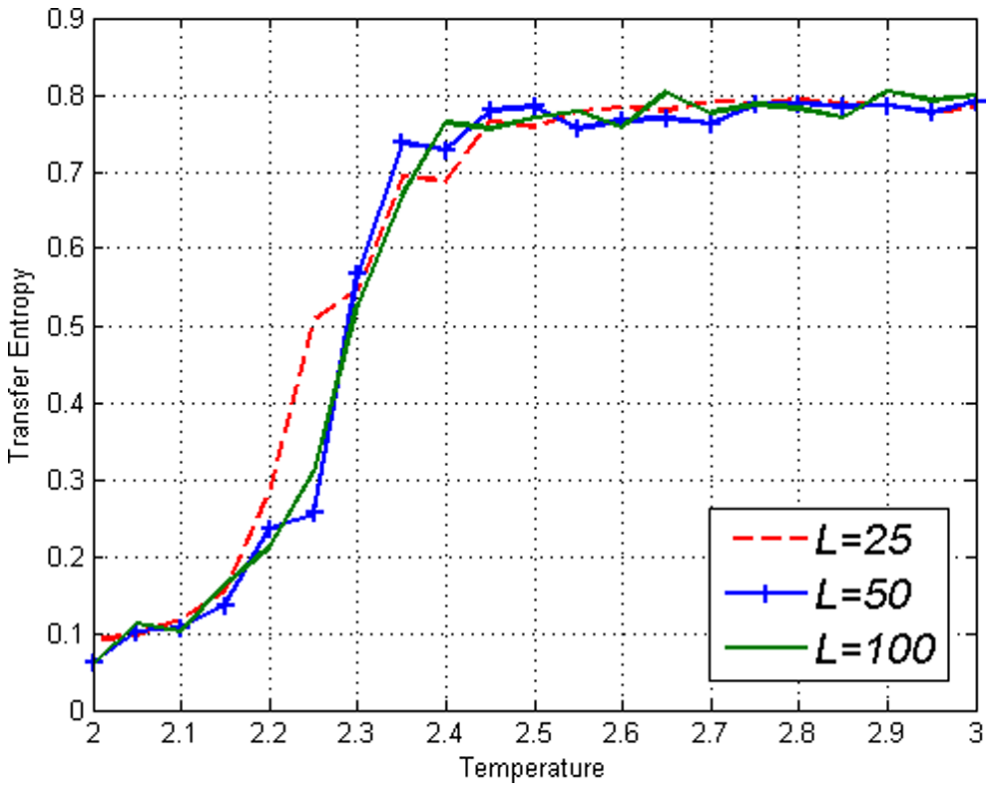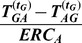 on amended Ising model with 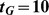 and 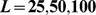. All with phase-transition like jump.

In order to understand the dynamics of of each site we calculate the effective rate of change (ERC) in relation to the transition probabilities. Let 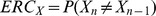 for any site 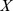 on the lattice. Figure (18) illustrates how 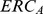 and 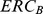 are equal, as expected, and significantly different from 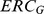. In Figure (10), the corresponding Transfer Entropy in both directions are displayed. At higher temperatures, it can be clearly seen that 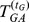 is larger than 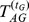. However for temperatures near 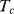 it is not as clear and therefore to highlight the relative values we calculate 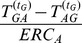 in Figure (19) and Figure (20) where 
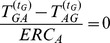
 if 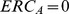. We see that this value actually gives a clear jump at 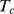 and remains more or less a constant after 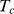. Therefore even though Transfer Entropy in neither direction is zero, a clear indication of directionality can be obtained. Interestingly, the division with ERC brought out the clear phase transition-like behaviour that seems to distinguish the situation below and above 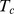. Referring back to Figure (4) of the unamended Ising model we can clearly see that 
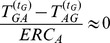
 for any direction in the unamended Ising model. We have demonstrated that 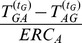 is able to cancel out the symmetric contribution from the collective behaviour and only captures the imposed directed interdependence.

In his introductory paper [16], Schreiber warns that in certain situations due to different information content as well as different information rates, the difference in magnitude should not be relied on to imply directionality unless Transfer Entropy in one direction is 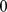. We have shown that when collective behaviour is present on the Ising model, the value of Transfer Entropy cannot possibly be 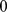. We suggest that this is due to fact that collective behaviour is as a type of ‘causality’ (disseminating information in all directions) and thus the Transfer Entropy is correctly indicating ‘cause’ in all directions. The clear difference in Transfer Entropy magnitude (even at 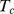) observed when the model is amended indicates that the *difference* in Transfer Entropy can indeed serve as an indicator of directionality in systems with emergent cooperative behaviour. We have seen that Transfer Entropy is influenced by the nearest neighbour interactions, collective behaviour and the ERC. In the next section we use the Random Transition model to further investigate how the ERC influences the Transfer Entropy.

## Random Transition Model

In the amended Ising model we implemented a causal lag as a restriction of one variable on another, in a way that a value of the source variable will affect the possible changes of the target variable. It is this novel concept of implementing ‘causality’ that we will analyze and expand in the Random Transition model. Let 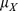, 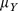 and 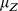, be the independent probabilities for the stochastic swaps of the variables 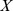, 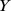 and 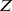 at every time step respectively. In addition to that, a restriction is placed on 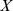 and 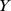 such that they are only allowed to do the stochastic swaps with probability 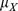 and 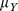 if the state of 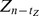 fulfills a certain condition. This restriction means that 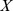 and 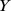 can only change states if 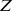 is in the conditioned state at time step 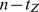 thus creating a ‘dependence’ on 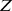, analogous to the dependence of 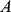 and 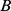 on 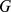 in the amended Ising model.

However in this model we allow the number of states 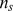 to be more than just two. The purpose of this is twofold, on one hand it contributes towards verifying that the behaviours of Transfer Entropy observed on the amended Ising model does extend to cases where 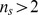. On the other hand, the model also serves to highlight different properties of Transfer Entropy as well as the very crucial issue of probability estimation that may lead to misleading results. The processes are initialized randomly and independently. The swapping probabilities are taken to be 
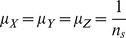
, thus enabling Transfer Entropy values to be calculated analytically. The transition probability of the Random Transition model is as follows. We assume that if a process chooses to change it must choose one of the other states equally, thus we have that 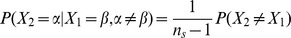, so that the marginal and joint probabilities remain uniform but the transition probabilities are
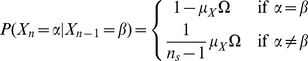


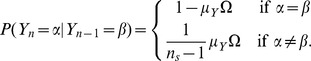
and
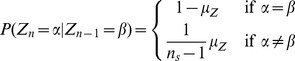
where 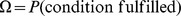 such that one can control ‘dependence’ on 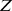 by altering 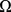.

### The relationship between 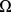 and 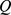


To understand how the values of 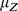 affects the value of 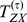 we need a different variable. Let 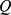 be the probability that the condition is fulfilled given current knowledge at time 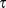 such that 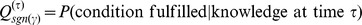. The value of 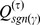 will depend on 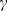, and in our model here, particularly on whether or not 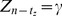 satisfies the condition. One can divide the possible states 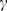 of all the processes into two sets such that
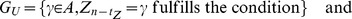


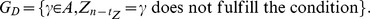
Note that 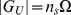 and 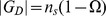 since 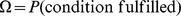 such that 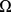 can be interpreted as the proportion of states of 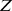 that fulfill the condition. Due to equiprobability of spins and uniform initial distribution, for any 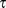 there are only two possible values of 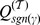, one for 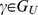 and one for 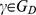. Therefore define 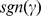 such that
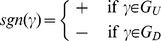
(11)to get
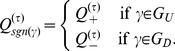
(12)Thus 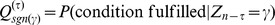 with the 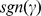 as in equation (11).

**Figure 21:**
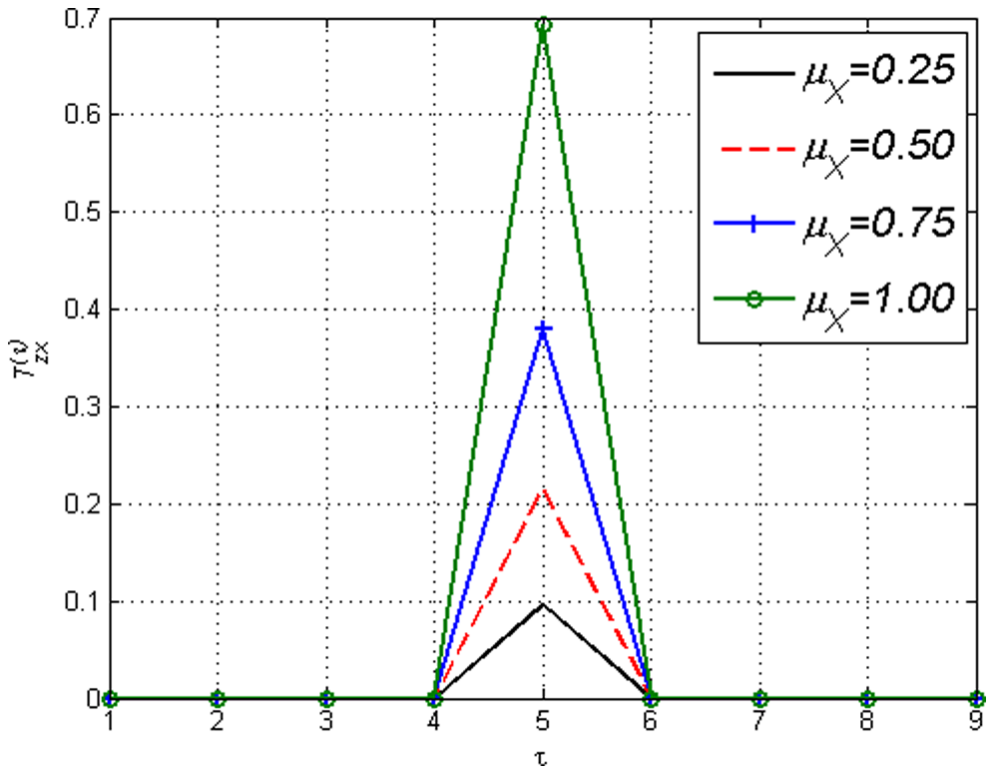
Analytical Transfer Entropy 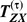 versus time lags 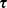 of the Random Transition model with 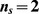 (hence 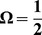) and 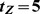 in equation (16) where 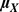 is varied but 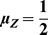 fixed. 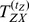
 is monotonically increasing with respect to 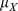. 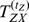 is affected by 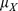. Figure illustrates how the internal dynamics of 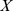 influences 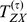 when 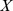 is the target variable. Transfer Entropy changes even though external influence 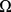 is constant.

The relationship between 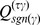 and 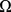 can be defined using the formula for total probability 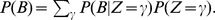 Let 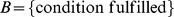 and using the fact that 
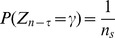
, we get that
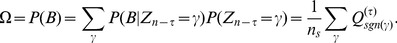
(13)Due to the sole dependence of 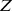 on 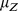, 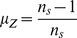 will make the transition probability of 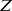 uniform such that 
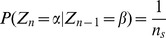
 for any 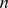 since we have that
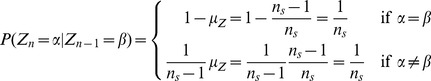
for any 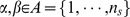. Consequently, 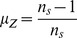 also makes all values of 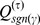 uniform so that equation (13) becomes
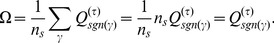
(14)Therefore on the model when the 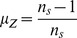, we have that 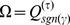 for any 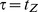. And this is why we get Figure (21), where 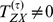 only if 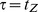 since 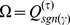 in equation (16) cancels out.

For any 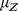, the relationship between 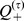 and 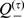 can be derived from equation (13) where
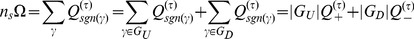
(15)

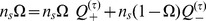


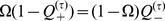
Note that when 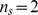 (hence 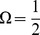) this simplifies to 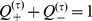.

### Transfer Entropy formula on the Random Transition model

Using 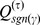 as in equation (12) we have that
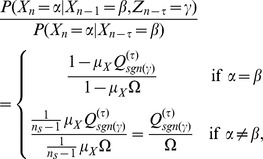
which gives us
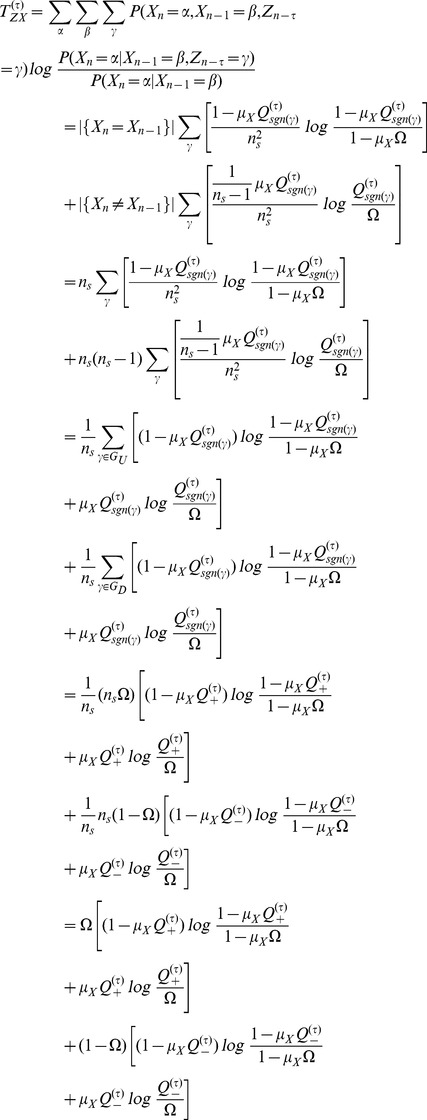
(16)where we used the Bayes theorem i.e
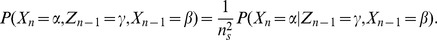
Due to independence, if 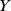 were to be conditioned on 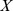 we would have that
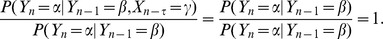
Therefore for values other than when 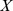 and 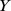 conditioned on 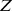, this ratio will yield 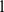. This renders 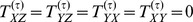. And if we get that 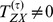, we can say that Transfer Entropy indicates ‘causality’ or some form of directionality from 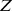 to 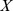 and 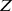 to 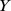, at time lag 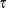. In a similar manner for 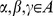 we have that
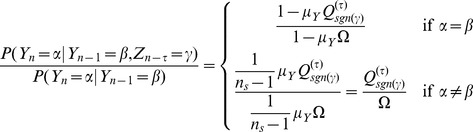
such that 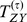 in exactly like equation (16) except that 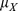 is replaced with 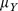.

When 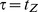 we have that 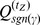 is either 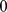 or 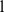 since the condition was placed at 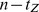. More specifically we will have that 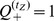 and that 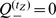. Putting these two values in equation (16) we obtain
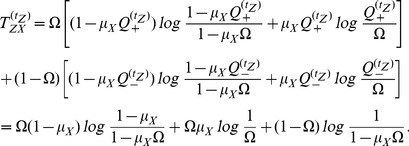
(17)A more thorough treatment of the Random Transition model and other methods of Transfer Entropy estimations is given in [32].

### Understanding ‘causality’ on the Random Transition model

The unclear meaning of the magnitude of Transfer Entropy is one of its main criticism [6], [18]. This is partly due to the ERC which incorporates both external and internal influences, the separation of which is rather unclear. The advantage of investigating Transfer Entropy on the Random Transition model is that the ERC can be defined in terms of internal and external elements i.e. for any variable 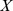 we have that
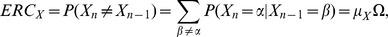
where 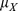 is the internal transition probability of 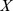 and 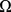 represents the external influence applied on 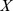. If the condition in our model is that 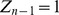 for 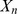 and 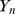 to change values then, 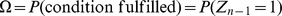 so that 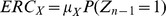 and 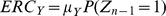. However, for the source 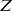 which has no external influence, 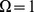 and consequently 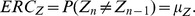


**Figure 22:**
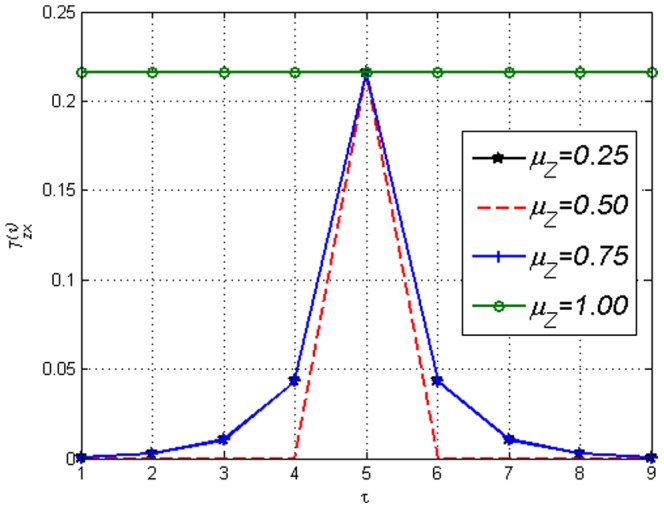
Analytical Transfer Entropy 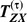 versus time lags 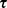 of the Random Transition model with 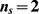 (hence 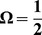) and 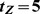 in equation (16) where 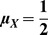 fixed and 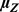 is varied. Only at 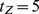, 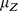 does not effect 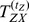 and values remain constant. For 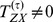 at 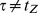, Transfer Entropy is affected by 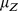. 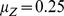 and 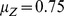 coincides. Figure shows how the internal dynamics of 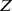 influences 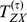 when 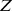 is the source variable.

When 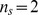, the model essentially replicates the Ising model without the collective behaviour effect i.e. far above the 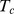 where the Boltzmann distribution approaches a uniform distribution. Consequently, at these temperatures the influence of collective behaviour is close to none. One can see in Figure (21) and Figure (22) that the 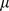 (hence the ERC) values are indeed key in determining the strength of Transfer Entropy. In Figure (21), 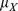 influences 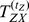 monotonically when every other value is fixed, therefore in this case the Transfer Entropy reflects the internal dynamics 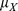 rather than the external influence 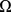. If ‘causality’ is the aim, surely 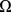 is the very thing that makes the relationship ‘causal’ and should be the main focus. This is a factor that needs to be taken into account when comparing the magnitudes of Transfer Entropy. Figure (21) also shows that when 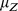 is uniform (since 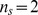) hence 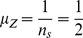, one gets that 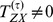 only if 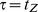 which makes causal lag detection fairly straight forward. However, in Figure (22) the effect of varying 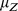 can be clearly seen in the nonzero values 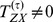 when 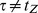. Nevertheless, the value at 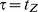 seems to be fully determined by 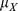 regardless of 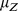 value. The mechanism in which 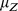 effects 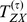 is sketched in the appendix.

Therefore one can conclude that when 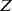 is the source (‘causal’ variable) and 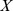 is the target (the variable being affected by the ‘causal’ link), the value of the Transfer Entropy 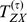 at 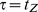 is influenced only by 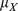 but for 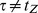, 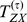 is determined by both 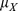 and 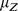. We have verified that this is indeed the case even when 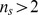 in this model. This should apply to all variables in the model and much more generally to any kind of source-target ‘causal’ relationship in this sense. We suspect that this also extends to cases when there is more than one source and this will be a subject of future research. Thus for causal lag detection purposes, it is clear that theoretically Transfer Entropy will attain maximum value at the exact causal lag. It is also clear that Transfer Entropy at nearby lags can be nonzero due to this single ‘causal’ relationship. Thus, on data sets it is strongly recommended to test for relative lag values.

### Transfer Entropy estimations of the Random Transition model

**Figure 23:**
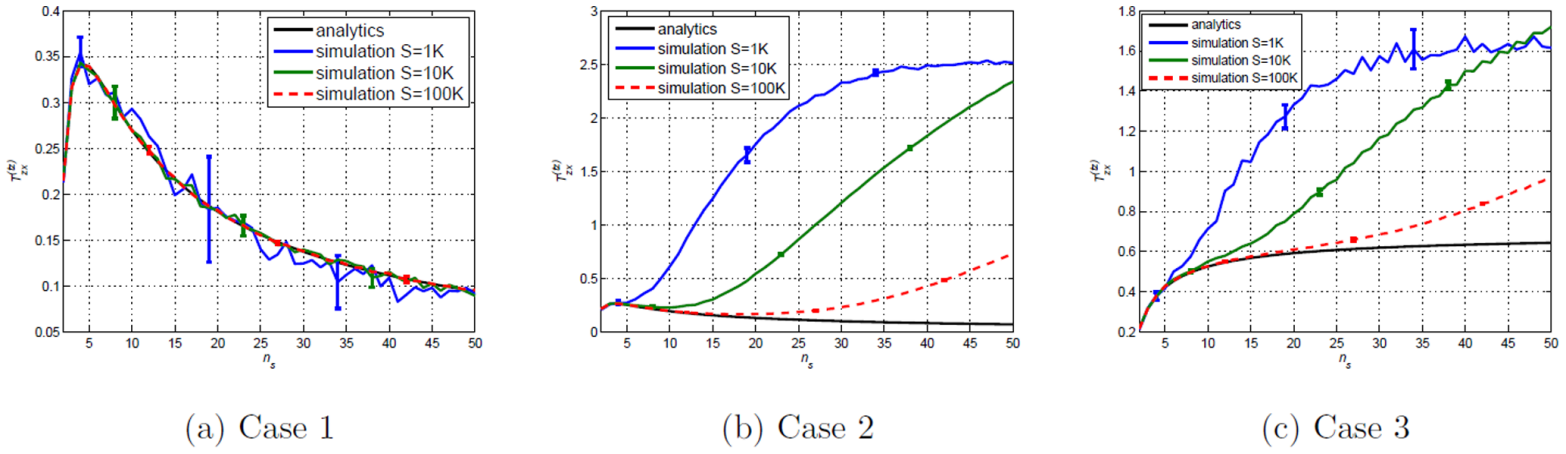
Transfer Entropy 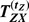 versus number of state 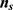 (number of chosen bins) for Cases 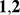 and 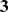. 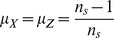
 are uniformly distributed. Analytical values obtained from substituting respective 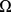 values in equation (17). Simulated values are acquired using equation (5) on simulated data of varying sample size 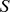 (length of time series) where 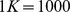. Error bars are displaying two standard deviation values above and two standard deviation below (some bars are very small, it can barely be seen). The aim is primarily to display how choosing 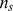 has to be made according to length, 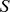, of available time series. For large 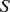 the error bar becomes smaller than the width of the curve.

For a classical histogram estimation of Transfer Entropy on real data sets [17], one can say that the number of states 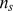 corresponds to the number of bins chosen for estimation. The estimations of Transfer Entropy for larger 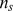 requires sufficient sample size (sufficient length of time series). To illustrate this finite sampling effect we set the value 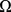 to three different values; 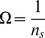 for Case 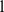, 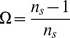 for Case 2 and 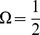 for Case 3. We plot the analytical Transfer Entropy 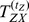, and its estimations on simulated values of varying time series length, 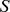, for all three cases in Figure (23). The exact 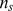 is known and incorporated in the estimations.

The observed existence of spurious detection or overestimation (finite sampling effects) as in Figure (23), is not uncommon and has been reported in relation to causality measures [15], [20], [33], [34]. This situation would be even more confusing in situations where 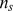 is not known (unfortunately, this is more often than not the case). The significant testing (or lack of it) of Transfer Entropy is admittedly one of its main criticism. Initially, we have sidestepped this issue by implementing Transfer Entropy on relatively small 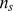 to easily get statistically significant estimations. In fact of the main motivation for the use of the Ising model in the testing of Transfer Entropy is to exactly sidestep this issue since no binning is required and one can focus on the issue of what exactly does the Transfer Entropy measures. However Figure (23) clearly shows that for larger 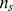, some form of validation is required to avoid false directionality conclusion. Surrogates have been suggested as a form of significant testing for Transfer Entropy [13], [20], [26], [35]. Surrogate data sets are synthetically generated data which should ideally preserve all properties of the underlying system except the one being tested [20]. There are many different types of surrogates to serve different purposes[13], [14], [16], [20], [26], [35]. The idea is to break the coupling (causal link) but maintain dynamics in hope that one can differentiate cause and effect from any other dynamics.

**Figure 24:**
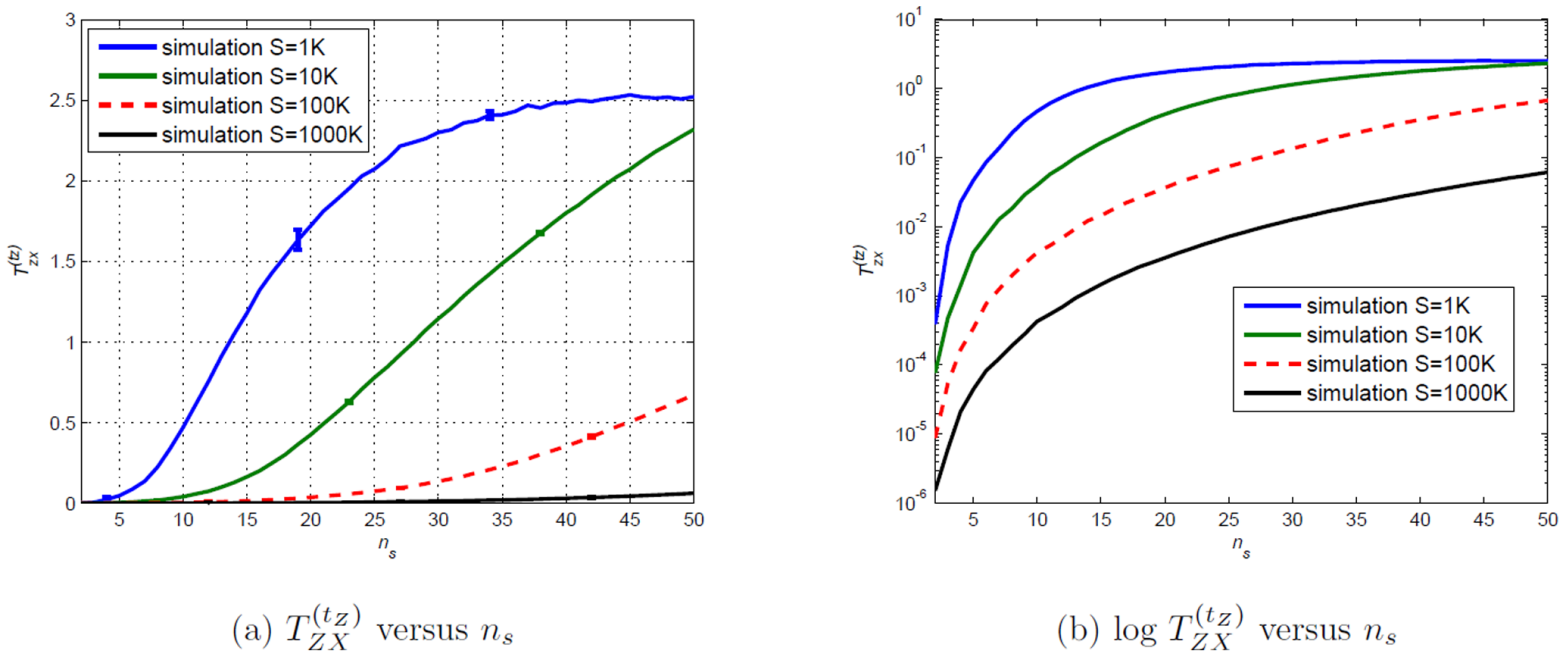
Transfer Entropy using equation (17) on simulated null model with varying sample size or length of time series, 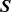 where 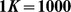. Analytical values are all 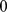. Error bars in the first figure are displaying two standard deviation values above and two standard deviation below. For large 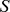 the error bar becomes smaller than the width of the curve. In order to use the null model as surrogates, 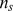 still has to be chosen in accordance to 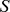.

One way to attain surrogates is by generating a null model (in the case of the Random Transition model this is simply three randomly generated time series) and test the values of Transfer Entropy as in Figure (24). Subtracting the null model from the values on the Random Transition model is equal to subtracting the Transfer Entropy values of both directions as one direction is theoretically zero. This is the idea behind the effective and corrected Transfer Entropy [15], [18]. However this does not quite solve the problem as the values may still be negative if the sample size is small. There are many other types of corrections [6], [13] proposed to address this issue involving substraction of the null model in some various forms. Nevertheless, as we have seen in Figure (19) of the amended Ising model, only by subtracting the two directions of Transfer Entropy did we obtain the clear direction as this cancelled out the underlying collective behaviour. We suspect that this will work as well for cancelling out other types of background effects and succeed in revealing directionality.

## Discussion

This paper highlights the question of distinguishing interdependencies induced by collective behaviour and individual (coupled) interactions, in order to understand the inner workings of complex systems derived from data sets. These data sets are usually in the form of time series that seem to behave essentially as stochastic series. It is hence of great interest to understand measures proposed to be able to probe ‘causality’ in view of complex systems. Transfer Entropy has been suggested as a good probe on the basis of its nonlinearities, exploratory approach and information transfer related interpretation.

To investigate the behaviour of Transfer Entropy, we studied two simplistic models. From results of applying Transfer Entropy on the Ising model, we proposed that the collective behaviour is also a type of ‘causality’ in the Wiener-Granger framework but highlighted that it should be identified differently from individual interactions by illustrating this issue on an amended Ising model. The collective behaviour that emerges near criticality may overshadow the intrinsic directionality in the system as it is not detected by measures such as covariance (correlation) and Mutual Information. We showed that by taking into account both directions of Transfer Entropy on the amended Ising model, a clear direction can be identified. In addition to that, we verified that the Transfer Entropy is indeed maximum at the exact causal lag by utilizing the amended Ising model.

By obtaining the phase transition-like *difference* measure, we have shown that the Transfer Entropy is highly dependent on the effective rate of change (ERC) and therefore likely to be dependent on the overall activity level given by, say, the temperature in thermal systems as we demonstrated in the amended Ising model. Using the Random Transition model we have illustrated that the ERC is essentially comprised of internal as well as external influences and this is why Transfer Entropy depicts both. This also explains why collective behaviour on the Ising model is detected as type of ‘causality’. In complex systems where there is bound to be various interactions on top of the emergent collective behaviour, the situation can be difficult to disentangle and caution is needed. Moreover we pointed out the danger of spurious values in the estimation of the Transfer Entropy due to finite statistics which can be circumvented to a certain extend by a comparison of the amplitude of the causality measure in both directions and also by use of null models.

We believe that identifying these influences is important for our understanding of Transfer Entropy with the aim of utilising its full potential in uncovering the dynamics of complex systems. The mechanism of replicating ‘causality’ in the amended Ising model and the Random Transition model may be used to investigate these ‘causality’ measures even further. Plans for future investigations involve indirect ‘causality’, multiple sources and multiple targets. It would also be interesting to understand these measures in terms of local and global dynamics in dynamical systems. It is our hope that these investigations will help establish these ‘causality’ measures as a repertoire of measures for complex systems.
